# The potential impact of climate change on the transmission risk of tick-borne encephalitis in Hungary

**DOI:** 10.1186/s12879-019-4734-4

**Published:** 2020-01-13

**Authors:** Kyeongah Nah, Ákos Bede-Fazekas, Attila János Trájer, Jianhong Wu

**Affiliations:** 1 Laboratory for Industrial and Applied Mathematics, Department of Mathematics and Statistics, York University, 4700 Keele St., Toronto, M3J 1P3 Canada; 2 MTA Centre for Ecological Research, Institute of Ecology and Botany, Alkomány u. 2-4., Vácrátót, H-2163 Hungary; 3 MTA Centre for Ecological Research, GINOP Sustainable Ecosystems Group, Klebelsberg Kuno u. 3., Tihany, H-8237 Hungary; 4 Institute of Environmental Engineering, University of Pannonia, Egyetem u. 10., Veszprém, H-8200 Hungary; 5 Department of Limnology, University of Pannonia, Egyetem u. 10., Veszprém, H-8200 Hungary

**Keywords:** Tick-borne encephalitis, Climate change, Non-systemic transmission, Summative indices

## Abstract

**Background:**

Impact of climate change on tick-borne encephalitis (TBE) prevalence in the tick-host enzootic cycle in a given region depends on how the region-specific climate change patterns influence tick population development processes and tick-borne encephalitis virus (TBEV) transmission dynamics involving both systemic and co-feeding transmission routes. Predicting the transmission risk of TBEV in the enzootic cycle with projected climate conditions is essential for planning public health interventions including vaccination programs to mitigate the TBE incidence in the inhabitants and travelers. We have previously developed and validated a mathematical model for retroactive analysis of weather fluctuation on TBE prevalence in Hungary, and we aim to show in this research that this model provides an effective tool for projecting TBEV transmission risk in the enzootic cycle.

**Methods:**

Using the established model of TBEV transmission and the climate predictions of the Vas county in western Hungary in 2021-2050 and 2071-2100, we quantify the risk of TBEV transmission using a series of summative indices - the basic reproduction number, the duration of infestation, the stage-specific tick densities, and the accumulated (tick) infections due to co-feeding transmission. We also measure the significance of co-feeding transmission by observing the cumulative number of new transmissions through the non-systemic transmission route.

**Results:**

The transmission potential and the risk in the study site are expected to increase along with the increase of the temperature in 2021-2050 and 2071-2100. This increase will be facilitated by the expected extension of the tick questing season and the increase of the numbers of susceptible ticks (larval and nymphal) and the number of infected nymphal ticks co-feeding on the same hosts, leading to compounded increase of infections through the non-systemic transmission.

**Conclusions:**

The developed mathematical model provides an effective tool for predicting TBE prevalence in the tick-host enzootic cycle, by integrating climate projection with emerging knowledge about the region-specific tick ecological and pathogen enzootic processes (through model parametrization fitting to historical data). Model projects increasing co-feeding transmission and prevalence of TBEV in a recognized TBE endemic region, so human risk of TBEV infection is likely increasing unless public health interventions are enhanced.

## Background

TBE, endemic in several Central and Eastern European countries including Hungary [1], is a tick-borne infectious disease caused by tick-borne encephalitis virus (TBEV), a member of the genus Flavivirus. Small mammals including rodents are the main reservoir hosts of the disease and humans are the accidental hosts. Human infection of TBEV involves the central nervous system [2].

In a previous study [3], we have developed a TBEV transmission dynamics model coupled with an integration of (infected) tick-human contacts during a surveillance interval to describe the tick population dynamics and TBEV transmission dynamics in the tick-host enzootic cycle and tick-human contact. Key ecological and epidemiological parameters, region- and environment-specific, were estimated by fitting weekly human incidence data and incorporating local temperature data in the Vas county of Hungary. The parameterized model was then used to conduct a retroactive assessment of the TBEV transmission patterns in the tick-host enzootic cycle in Hungary to conclude that the prevalence of TBEV transmission in the enzootic cycle had been increasing along with the observed warming weather. This study also confirmed that non-systemic (co-feeding) transmission, whereby a susceptible (larva or nymph) can acquire infection from co-feeding with infected nymphs in the same host even when the infection has not been established in the host [4], has been playing a very significant role in maintaining the transmission cycle of TBEV in Hungary and nearby regions [3, 5, 6]. Similar observations were made using a few early mechanistic models which incorporated the non-systemic transmission route [7–9]. Although the transovarial transmission of TBEV is also possible, it is considered not efficient for maintaining the pathogen in tick populations [10, 11], and so it was not included in the model. Here, we aim to use this validated model to assess the impact of climate change on the TBE infection risk in the tick-host enzootic cycle. As discussed in [12], climate change may impact vector-borne disease transmission in many different ways including the change of the transmission intensity or the duration of the transmission season.

An early study [13] provided ample evidence for the significance of TBE co-feeding transmission in Europe, and this fundamental study also showed that projected climate change would result in the contraction of the range of TBEV in some part of the Europe by lowering the degree of coincident feeding of larval and nymphal ticks [13]. However, whether this climate change increases or decreases the TBE prevalence in the tick-host enzootic cycle in a particular region depends highly on the patterns of the projected region-specific climate change.

Here, we aim to incorporate the climate change patterns into the TBEV transmission dynamics model by revising model parameters, to project the TBEV prevalence in the tick-host enzootic cycle, and evaluate whether co-feeding and systemic transmission combined can sustain the persistence of TBEV. Our results show that in the study region, Vas County, the climate change will double and triple several key summative indices measuring the reproduction ratio (the basic reproduction number, *R*_0_), stage-specific tick densities, duration of co-feeding period, co-feeding ticks at two physiological stages (larva and nymph), and accumulated (tick) infections per host due to co-feeding transmission, during 2021-2050 and 2071-2100 according to two different climate predictions.

## Methods

### Study area and climate data

**Fig. 1:**
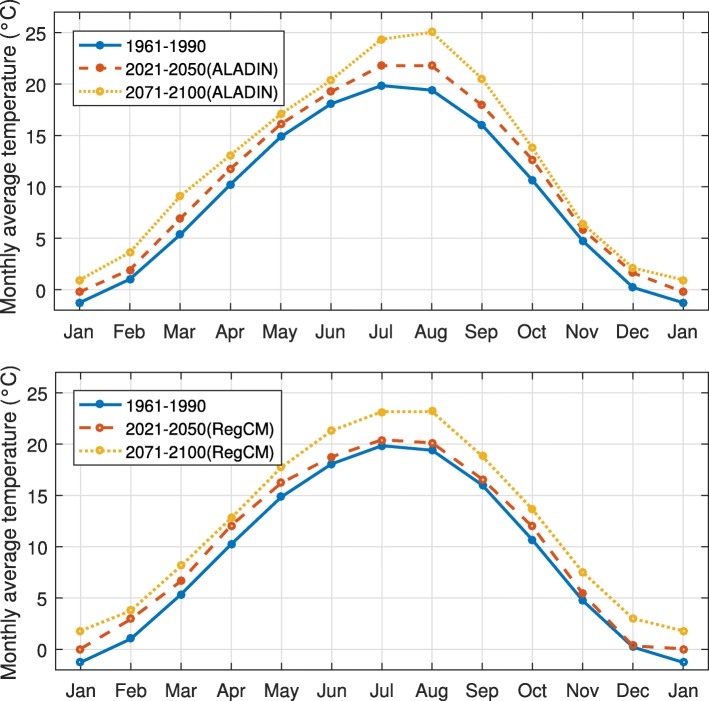
Observed and predicted climate data. The blue curve shows the monthly mean temperature values observed during 1961–1990 (CarpatClim-Hu database) and the red and yellow curves show the predicted monthly mean temperature in 2021–2050 and 2071–2100, respectively (Top panel: ALADIN-Climate 4.5, Bottom panel: RegCM 3.1). The corresponding coordinate of the database is 47.2^∘^N 16.6^∘^E

We used monthly average temperature values observed in the past (1961-1990) and the values predicted in the future times, during the period of 2021-2050 and 2071-2100 (Fig. 1). The coordinate of the grid point is 47.2^∘^N and 16.6^∘^E, which is the nearest located to the center of Szombathely in Vas county, one of the main endemic areas in Hungary [14, 15]. For the climate data in 1961–1990, we used CarpatClim-Hu database [16]. For the future periods, we used two regional climate models (ALADIN-Climate 4.5 [17] and RegCM 3.1 [18, 19]) driven by the A1B emission scenario of IPCC SRES [20, 21] which describes the radiative forcing of 850 ppm CO_2_ concentration by 2100 [22].

### Tick-borne encephalitis virus transmission dynamics

**Fig. 2:**
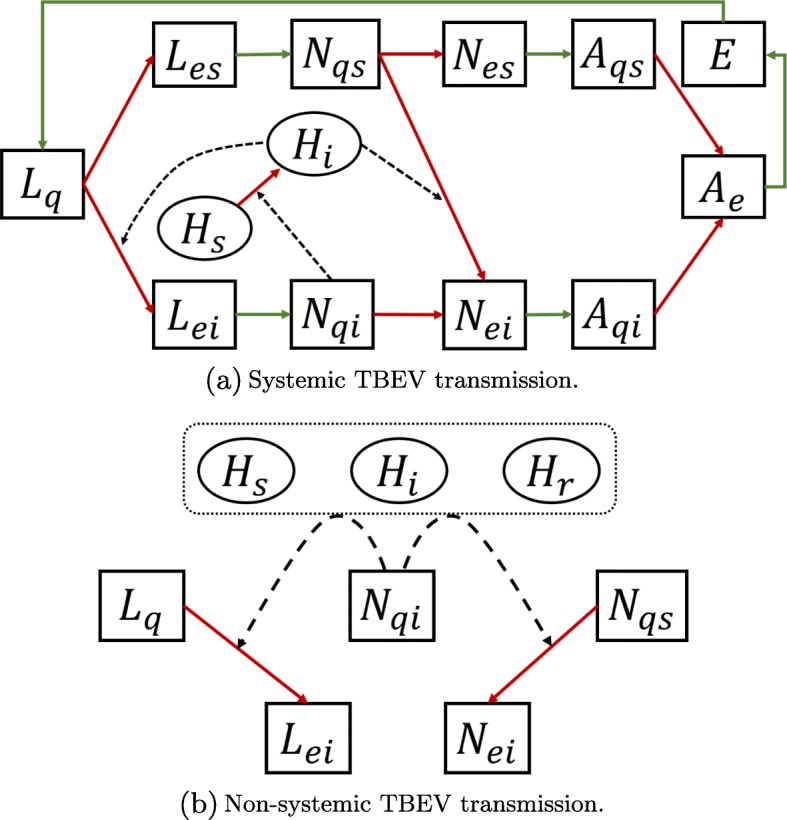
A schematic diagram of TBEV transmission. **a** represents the flow of TBEV transmission through systemic transmission route. The dashed lines represent systemic infection routes. The transitions depicted by solid lines are seasonal and parameterized by temperature variable. The green lines represent development rate from eggs to larvae, engorged stages to the next stages and the oviposition rate. The transitions in red lines are determined by host-attaching rates which are described by seasonal host availability and seasonal tick-questing activities. The diagram in (**b**) shows non-systemic transmission of TBEV induced by co-feeding of susceptible ticks (*L*_*q*_, *N*_*qs*_) and infected nymphs (*N*_*qi*_) on the same hosts, regardless of the infection status of hosts. The dashed lines represent non-systemic infection routes

The model in [3] describes the seasonal transmission of TBEV in the enzootic cycle with ticks and (animal) hosts. Two major transmission routes are considered: the systemic transmission route (Fig. 2a) and the non-systemic transmission route (Fig. 2b).

In this model, the tick population is stratified as eggs (*E*), questing larvae (*L*_*q*_), engorged larvae (*L*_*e*_), questing nymphs (*N*_*q*_), engorged nymphs (*N*_*e*_), questing adults (*A*_*q*_) and engorged adults (*A*_*e*_). The tick population growth is described by the following parameters: birth rate of eggs, host-attaching rates, survival probability of the host-attached ticks, development rates from engorged states to the next stages and the mortality rate at each stage. The development rates and host-attaching rates are temperature dependent parameters. The host-attaching rates depend on the proportion of actively questing ticks and the host availability at the temperature. Although we have parameterized the questing activity of ticks by temperature, it is suggested that the day length and the air humidity also affect the questing activity [23].

The model considers systemic transmission among immature ticks and hosts when susceptible questing ticks (*L*_*q*_ or *N*_*qs*_) bite infected hosts (*H*_*i*_), or when infected questing nymphs (*N*_*qi*_) feed on the susceptible hosts (*H*_*s*_). The systemic transmission dynamics is governed by the transmission efficacy from hosts to larvae, hosts to nymphs, nymphs to hosts and the recovery rate of the infected host, and it is implicitly influenced by the natural population growth of ticks and hosts and the host-attaching rates.

In order to quantify the risk at which a susceptible feeding tick is infected by co-feeding nymphs, the model includes the following additional parameters: average duration of feeding for infected nymphs, probability of an infected nymph to induce non-systemic infection to the co-feeding susceptible ticks.

### Retroactive and proactive assessment of transmission risk in the enzootic cycle

Using this transmission dynamics model, we invented a few key summative indices to quantify the regional risk of TBEV transmission: the yearly duration of questing activities (1-dimensional index for feeding duration), the area bounded by tick density curves (ABC) (2-dimensional index for “force of co-feeding"), and the multiplication of ABC with the peak of infected questing nymphs (3-dimensional index for accumulated infection due to co-feeding). We also use the basic reproduction number to measure the transmission power of TBEV in the region with the specific environmental conditions. We define these indices in detail below.

**Yearly duration of questing activity for larvae,*T***_***l***_**:** As the unfed larvae are actively questing at time *t* only when the temperature at the time, *T*(*t*), is greater than *m*_*l*_, the minimum temperature for the coincidence of host availability and the activity of questing larvae, we define the yearly duration of questing activity for larvae (*T*_*l*_) by

$$T_{l}=\int_{0}^{365} H(T(t)-m_{l})dt /365, $$

where the time *t*=0 corresponds to the beginning of the year, and *H* is the Heaviside step function. Unit time is one day. *T*_*l*_ is the maximum length of the time window when larvae can possibly be infected by feeding an infected host (systemic transmission) or by co-feeding a host with infected nymphs (non-systemic transmission).

**Yearly duration of questing activity for nymphs,*T***_***n***_**:** This is defined in a similar way as for larvae:

$$T_{n}=\int_{0}^{365} H(T(t)-m_{n})dt /365. $$

Note that the minimum of *T*_*l*_ and *T*_*n*_ represents the duration of possible non-systemic transmission by co-feeding of infected nymphs and susceptible larvae.

**ABC of unfed larvae during questing season,*A***_***l***_**:** It is measured by the area under the density curve of unfed larva which are actively questing;

$$A_{l}=\int_{0}^{365} p_{l}(T(t))L_{q}(t)dt/365, $$

where *L*_*q*_(*t*) is the number of unfed larvae per host at time *t* and *p*_*l*_(*T*) is the probability of an unfed larvae to be actively questing at temperature *T*, modelled by *p*_*l*_(*T*)=*H*(*T*−*m*_*l*_). All unfed larvae are susceptible and the higher value of *A*_*l*_ represents the larger number of susceptible questing larvae within a season.

**ABC of unfed susceptible nymphs during questing season,*A***_***ns***_**:**

$$A_{ns}=\int_{0}^{365} p_{n}(T(t))N_{qs}(t)dt/365, $$

where *p*_*n*_(*T*)=*H*(*T*−*m*_*n*_). The greater *A*_*ns*_, the more number of susceptible nymphs are actively questing in a season.

**ABC of susceptible questing ticks times the peak of infected questing nymphs,*V***_***c***_**:** This is defined by

$$V_{c}=\overline{N_{qi}}(A_{l}+A_{ns}), $$

where $\overline {N_{qi}}$ is the maximum density of infected questing nymphs within a year. Higher value of *V*_*c*_ means higher chance of non-systemic transmission triggered by co-feeding of infected nymphs and susceptible ticks.

**Basic reproduction number,*R***_**0**_**:** The basic reproduction number is used to measure the rate at which the number of TBEV infected ticks grow. Assuming that the TBEV transmission model is a periodic system with one year period, we compute the three values of basic reproduction numbers using the monthly average climate data of 1961–1990, 2021–2050 and 2071–2100, respectively.

### Estimation for the contribution of non-systemic transmission

We quantify the significance of non-systemic transmission in TBEV transmission in a year by computing the expected number of TBEV transmission to ticks per host via systemic and non-systemic transmission route. The expected number of transmission via systemic (non-systemic) transmission route is obtained by integrating the force of infection from infected hosts (infected co-feeding ticks). To implement this, we first obtained the numerical solutions of the system and then estimated the cumulative force of infection using trapezoidal numerical integration.

## Results

**Fig. 3:**
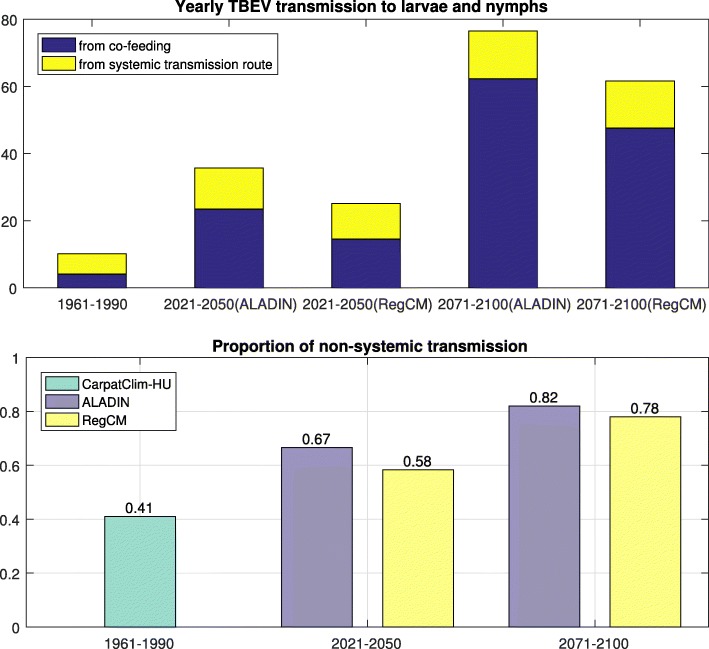
Significance of non-systemic transmission route in the transmission of TBEV. The top panel shows the yearly number of TBEV transmission to larvae and nymphs per host. The blue (yellow) color represents the transmission through non-systemic (systemic) transmission route. The proportion of non-systemic transmission is plotted in the lower panel

The results in Fig. 3 show that TBEV infection in the tick population increases in the future times, and that the contribution of co-feeding transmission to the overall transmission increases. In particular, it is estimated that 41% of TBEV transmission in ticks is induced through the non-systemic transmission route during 1961-1990. In 2021-2050 (2071-2100), the non-systemic transmission route is estimated to be responsible for 67% (82%) of the total transmission with the data from ALADIN database.

**Fig. 4:**
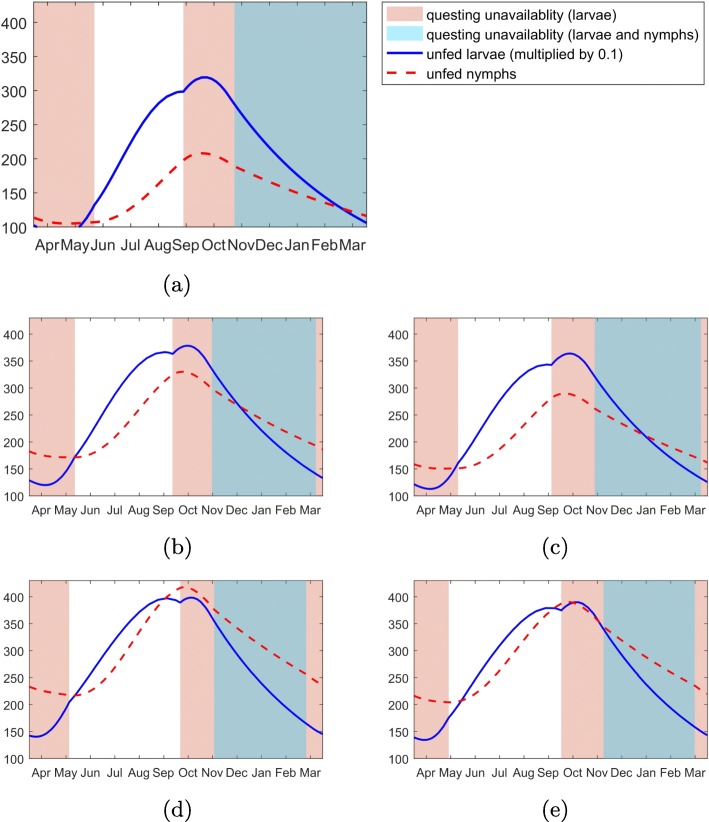
Seasonal questing behavior of larvae and nymphs. The red (blue) curve represents the number of unfed nymphs (10% of unfed larvae population) per host. The red (blue) region corresponds to the time period when the unfed larvae (nymphs) are not available for questing, either due to the behavioral diapause or the host unavailability. **a** 1961-1990, CarpatClim-HU. **b** 2021-2050, ALADIN. **c** 2021-2050, RegCM. **d** 2071-2100, ALADIN. **e** 2071-2100, RegCM

As shown in Fig. 4, the density of questing ticks is expected to rise. In particular, the density of questing nymphal ticks is expected to increase dramatically, resulting in the increase of co-feeding transmission as observed in Fig. 3.

**Fig. 5:**
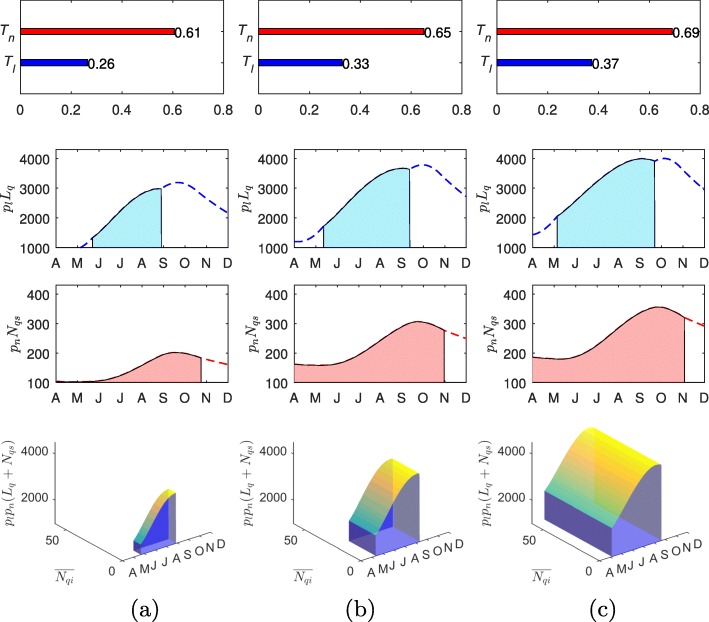
Transmission risk of TBEV projected with ALADIN model. The top panels, mid-panels and the low panels show the yearly duration of questing activity for larvae (*T*_*l*_) and nymphs (*T*_*n*_), the area under the curves of unfed larvae (*A*_*l*_) and susceptible nymphs (*A*_*ns*_), and the volume representing the chance of non-systemic transmission triggered by co-feeding of infected nymphs and susceptible ticks (*V*_*c*_), respectively. The panels in the left, middle and right side are produced using CarpatClim-HU and ALADIN climate data in 1961-1990, CarpatClim-HU 2021-2050 and 2071-2100, respectively. **a** 1961-1990. **b** 2021-2050, ALADIN. **c** 2071-2100, ALADIN

**Fig. 6:**
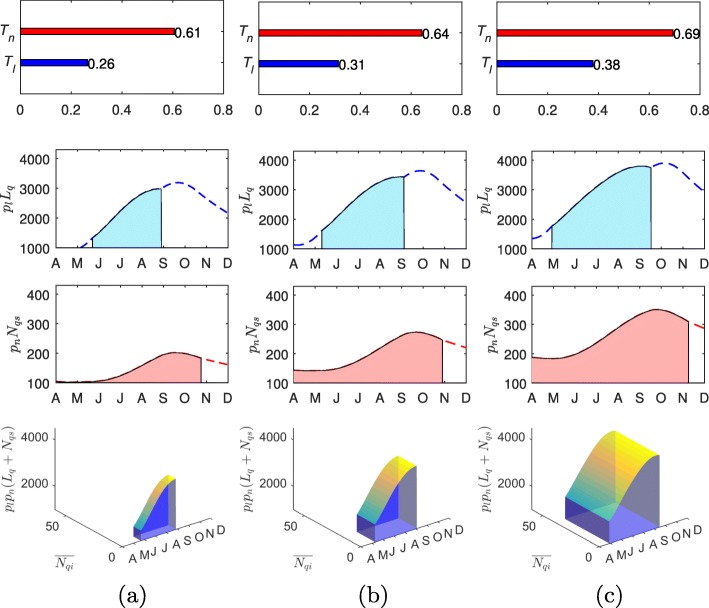
Transmission risk of TBEV projected with RegCM model. The top panels, mid-panels and the low panels show the yearly duration of questing activity for larvae (*T*_*l*_) and nymphs (*T*_*n*_), the area under the curves of unfed larvae (*A*_*l*_) and susceptible nymphs (*A*_*ns*_), and the volume representing the chance of non-systemic transmission (*V*_*c*_), respectively. The panels in the left, middle and right side are produced using CarpatClim-HU and RegCM climate data in 1961-1990, 2021-2050 and 2071-2100, respectively. **a** 1961-1990, CarpatClim-HU. **b** 2021-2050, RegCM. **c** 2071-2100, RegCM

The increasing transmission risk in the enzootic cycle can also be quantified, as shown in Figs. 5-6, using several indices we have introduced. For example, the panels in the 1 ^st^, 2 ^nd^ and 3 ^rd^ row show the yearly duration of questing activities (1-dimensional), area under the curves of questing ticks (2-dimensional) and the chance of non-systemic transmission triggered by co-feeding ticks (3-dimensional). More precisely, the panels in the first row show that the durations of questing activity of both larvae and nymphs increase. This leads to the increase of the duration when co-feeding between larval and nymphal ticks is possible, illustrated by panels in the 2nd row. Moreover, the increased temperature will shorten tick maturation delays and raise the number of the ticks. Finally, as shown in the 3rd row, the chance of non-systemic transmission will increase along with the temperature increase. The results are robust to the climate models used – ALADIN model and RegCM are used for the simulations of Figs. 5 and 6, respectively.

**Fig. 7:**
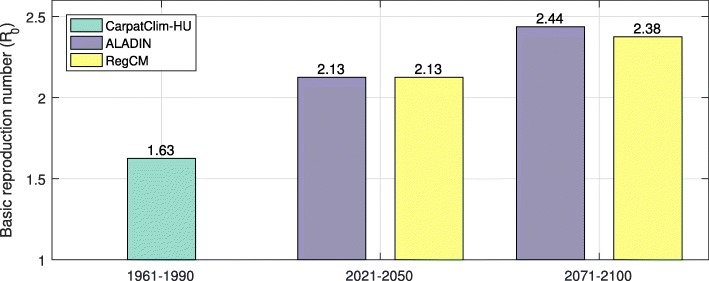
Transmission potential of TBEV. The bars indicate the basic reproduction numbers obtained from different climate models

The transmission potential is expected to rise as well. The basic reproduction number (*R*_0_) in 2021-2050 is expected to increase by 31% compared to the risk estimated for 1961-1990, and *R*_0_ in 2071-2100 is expected to increase by 50% (see Fig. 7).

## Discussion

TBE mainly occurs in the temperate climate countries [24]. *Ixodes ricinus*, the main vector transmitting TBEV in Europe occurs also in the Mediterranean countries [25]. The volume of the projected biome shifts in Europe indicates that the climate of Hungary will remain within the suitable range [26] and *Ixodes ricinus* ticks may still be present in Europe in the next decades. Using a mechanistic model parameterized through data fitting to historical human incidence data, we have produced estimates of future TBEV transmission risk in the tick-host enzootic cycle under projected climate change in an endemic region in Hungary. These estimations have been reported in terms of key summative indices - duration of questing activity, area under the curves of susceptible ticks and the degree of co-feeding transmission - in addition to the basic reproduction number. Our results show that the TBE infection risk in the tick-host enzootic cycle in the study region (Vas county, Hungary) will increase substantially, and our model-based analyses describe how this TBE infection risk increase results from compound factors including extended questing seasons, increasing co-feeding susceptible larval and nymphal ticks with infected nymphal ticks, and consequently increasing contribution of non-systemic transmission to sustain the transmission.

There have been some intensive attention to the impact of climate change on the TBE transmission in Europe. It was considered in [13] that the endemic area of TBEV in Europe will be reduced by the decreased level of co-feeding transmission. This study did note that the impact of the climate change on the transmission risk of TBE depends on the region-specific tick ecological and pathogen enzootic process which are highly affected by the region-specific climate change. Our study has shown that the transmission potential and the risk in the study site are expected to increase as well as the chance of transmission by co-feeding ticks. The extended length of tick season in Hungary is expected in the study [27]. Several other studies have also assessed the impact of climate change on the activities of ticks and the spread of tick-borne diseases [27–31], addressing the importance of the proactive action plans against the changing risk.

Recent fields observation studies show that saturation deficit is a better predictor of the density of questing ticks than the temperature alone [32, 33]. The density of ticks detected in nature is determined by various environmental factors including host availability. We have avoided to use those density data as an input parameter to our mechanistic model as the data is rarely available. A few laboratory experiments studied relationships between saturation deficit and some factors related to tick activities such as the duration of feeding and the quiescence [23]. These functional relationships could not be directly translated into the parameters of our model, however, in our previous study [3], several observation data were used to validate the parameterized model.

A limitation of this study is the implicit assumption of less change of the host abundance. The degradation and loss of forests and the northward migration of the hosts (deers, wild boars), catalyzed by climate change and human activities, may contribute to reducing the TBE prevalence in the enzootic cycle. We have restricted our study to the TBEV transmission in the enzootic cycle. Predicting its consequence in terms of human TBE incidence will need prediction of the human immunity acquired from vaccination and boosting programs, as well as the consumption of unpasteurized goat milk which can cause human TBE infection [34]. However, the projected increase in the TBE prevalence in the enzootic cycle suggests increasing public health investment to manage the consequence of climate change induced increase of TBEV.

## Conclusions

Transmission risk of TBEV in the enzootic cycle will increase in 2021-2050 and 2071-2100 in the study area as the climate change will facilitate the co-feeding transmission. Summative indices to quantify and explain this TBEV risk increase can be defined and calculated from a parametrized TBEV transmission model and using climate prediction data.

## Data Availability

All data generated or analysed during this study are included in this published article.

## Abbreviations

TBE: Tick-borne encephalitis
TBEV: Tick-borne encephalitis virus
